# Basic and Clinical Andrology reviewer acknowledgement 2015

**DOI:** 10.1186/s12610-016-0030-y

**Published:** 2016-03-03

**Authors:** Roger Mieusset

**Affiliations:** CHU Hôpital Paule de Viguier, 31300 Toulouse, France

## Abstract

The editor of *Basic and Clinical Andrology* would like to thank all of our reviewers who have contributed to the journal in Volume 25 (2015).

**Alex Varghese**

United States of America

**Antoine Bennet**

France

**Celia Ravel**

France

**Clement Jimenez**

France

**Csilla Krausz**

Italy

**Daniele Ribeiro**

Brazil

**Eberhard Nieschlag**

Germany

**Eric Huyghe**

France

**Florence Boitrelle**

France

**Glaura Fernandes**

Brazil

**Hans-Gert Bernstein**

Germany

**Harrison Pope Jr**

United States Minor Outlying Islands

**Hiram Gay**

United States of America

**Irma Scholten**

Netherlands

**Jacques Auger**

France

**Javier Angulo**

Spain

**Jean Parinaud**

France

**Jeanne Perrin**

France

**Joel Drevet**

France

**Louis Hermo**

Canada

**Luboslav Stárka**

Czech Republic

**Marc Garnick**

United States of America

**Maria Hernandez**

Mexico

**Maria-Christina Avellar**

Brazil

**Mário Sousa**

Portugal

**Marjan Sabbaghian**

Iran

**Omotayo Erejuwa**

Malaysia

**Pedro Fontes Oliveira**

Portugal

**Pierre Watcho**

Cameroon

**Rachel Guiton**

France

**Ricardo Bertolla**

Brazil

**Robert Given**

United States of America

**Safouane Hamdi**

France

**Sergey Moskovtsev**

Canada

**Sylviane Hennebicq**

France

**Thomas Ebner**

Austria

**Valerie Grandjean**

France

**Yvon Englert**

Belgium

